# Incidence, Predictors and Outcome of Vasoplegia After Left Ventricular Assist Device Implantation

**DOI:** 10.3390/jcdd13090456

**Published:** 2026-09-10

**Authors:** Zaki Haidari, Iskandar Turaev, Ender Demircioglu, Stephan Knipp

**Affiliations:** Department of Thoracic and Cardiovascular Surgery, West German Heart and Vascular Centre, University Hospital Essen, 45147 Essen, Germany

**Keywords:** heart failure, left ventricular assist device, cardiac surgery, vasoplegia

## Abstract

Background: Vasoplegia after heart failure surgery has been associated with increased morbidity and mortality. The aim of this study was to evaluate the incidence and impact of postoperative vasoplegia in patients undergoing durable continuous flow left ventricular assist device implantation (cfLVAD). Methods: All patients who underwent cfLVAD implantation were included in this analysis. Vasoplegia was defined as the need for norepinephrine ≥0.2 μg/kgBW/min and/or any vasopressin dose for ≥12 h starting within 72 h postoperatively without bleeding/pericardial tamponade requiring surgical revision. Endpoints included the incidence of vasoplegia and 30- and 90-day mortality. Cox proportional hazard model was used to assess the association between vasoplegia and mortality. Multivariate logistic regression analysis was performed to identify predictors of vasoplegia. Results: Between 08/2010 and 12/2023, 307 consecutive patients underwent durable cfLVAD implantation. Mean age was 58 ± 11 years. Of the patients, 94% were operated with cardiopulmonary bypass and in 71% intraoperative hemoadsorption was applied. The incidence of vasoplegia was 23%. Vasoplegia was significantly associated with increased short-term mortality compared to non-vasoplegic patients (HR: CI: *p* < 0.001). Multivariate regression analysis identified preoperative invasive ventilation and preoperative vasopressor support as strong risk factors for postoperative vasoplegia, while intraoperative hemoadsorption reduced this risk. Conclusion: Vasoplegia after durable cfLVAD implantation is a common and serious complication associated with increased short-term mortality. Preoperative mechanical ventilation and vasopressor support are risk factors for developing postoperative vasoplegia. Intraoperative hemoadsorption appears to reduce the risk of vasoplegia after durable cfLVAD implantation.

## 1. Introduction

Vasoplegia after cardiac surgery is characterized by systemic hypotension despite normal to increased cardiac output [1]. The underlying pathophysiologic mechanism leading to postoperative vasoplegia is complex and multifactorial. Proinflammatory cytokines trigger systemic inflammation due to the extracorporeal circulation and surgical trauma, which can lead to severe vasodilatation [2] that requires high vasopressor support and results in poor outcomes [3,4]. Heart failure (HF) patients undergoing cardiac surgery are at increased risk of developing postoperative vasoplegia [5] due to activated innate and adaptive immune systems and increased circulating concentrations of proinflammatory cytokines compared to healthy individuals [6]. Moreover, many of these HF patients already require mechanical circulatory support in the preoperative course, which further aggravates cytokine release [7]. This systemic inflammatory response is orchestrated in large part by high concentrations of circulating cytokines, a phenomenon referred to as “cytokine storm” in the most severe cases [8]. In HF patients undergoing durable continuous flow left ventricular assist device (cfLVAD) implantation, postoperative pre- and afterload sensitivities increase significantly. In combination with vasoplegia, it has major consequences on right ventricular function and outcome [9].

The aim of the present study was to evaluate the incidence and outcome of vasoplegia using a simplified definition in patients undergoing durable cfLVAD implantation and to identify the predictors of vasoplegia in this particular cohort of patients.

## 2. Methods

### 2.1. Patients

Patients who underwent cfLVAD implantation, either as an isolated procedure or in combination with other concomitant cardio-surgical procedures, were eligible for this study. The indication for durable cfLVAD was in accordance with the European Society of Cardiology guidelines [10,11] and the institutional heart failure protocol. The only exclusion criterion was that the cfLVAD was used as part of a total artificial heart approach. The study was performed in accordance with the Declaration of Helsinki, and the local ethics committee approved the study protocol (Protocol number: 22-10875-BO). Individual informed consent was waived.

### 2.2. Perioperative Management

All patients were operated under general anesthesia with endotracheal intubation and mechanical ventilation. Preoperative transesophageal echocardiography was performed to evaluate cardiac and valvular function. As per the institutional standard, isolated cfLVAD implantation was performed with cardiopulmonary bypass (CPB) in beating heart technique. Since 2014 and whenever feasible, intraoperative hemoadsorption using CytoSorb^®^ (Cytosorbents^©^, Princeton, NJ, USA) device was integrated into the CPB. In patients with indications for additional procedures, cold crystalloid cardioplegic arrest was performed when necessary.

Postoperatively, all patients were transferred to the cardiac surgery intensive care unit (ICU). Postoperative care consisted of routine invasive hemodynamic and pulmonary monitoring and guideline-based heart failure therapy. Inotropic support consisted of continuous administration of epinephrine and/or milrinone and/or dobutamine. Preoperative inotropic support was defined by any dose of the aforementioned inotropic infusion. Vasopressor treatment consisted of norepinephrine. Preoperative vasopressor need was defined as the norepinephrine requirement of ≥ 0.05 µg/kgBW/min. In cases requiring > 0.5 µg/kgBW/min norepinephrine, vasopressin was added. Standard antibiotic prophylaxis consisted of broad-spectrum antibiotic treatment for 7 days postoperatively. Anticoagulation consisted of intravenous heparin in addition to antiplatelet therapy with aspirin and/or clopidogrel, starting 24 h after surgery. Right ventricle (RV) failure prophylaxis or therapy consisted of early extubation, oral phosphodiesterase type 5 inhibitor and/or inhaled prostaglandin I2 analog and/or inhaled nitric oxide when indicated. In cases with therapy refractory RV failure, early (intraoperative or postoperative) mechanical RV support was considered. All patients underwent the LVAD education program before discharge. In addition, structured clinical follow-up performed by the institutional LVAD coordinator was part of the postoperative care.

### 2.3. Endpoint Definitions

The primary endpoints were the incidence of postoperative vasoplegia and all-cause mortality (30- and 90-day). Vasoplegia was defined as a requirement for norepinephrine ≥0.2 μg/kgBW/min and/or vasopressin at any dose for ≥12 h, starting within 72 h postoperatively after the index procedure. Postoperative vasopressor support greater than 0.2 µg/kg/min in patients with severe bleeding or pericardial tamponade requiring (operative) intervention was not classified as vasoplegia. Secondary endpoints included postoperative mechanical right ventricular (RV) support, new disabling stroke, prolonged ventilation, new renal replacement therapy (RRT), infections, length of intensive care and hospital stay. Disabling stroke was defined as stroke with a modified Rankin score >3. Prolonged ventilation was defined as postoperative need for mechanical ventilation longer than 21 days for at least six hours per day. New RRT was defined as the application of any new temporary or permanent RRT. Infection was defined as any new initiation, change, or escalation of antibiotic treatment beyond perioperative antibiotic prophylaxis during hospitalization.

### 2.4. Statistical Analysis

Statistical analysis was performed using SPSS Version 27 (SPSS Inc., Chicago, IL, USA). Continuous variables are expressed as mean and standard deviation (normally distributed) or median and interquartile range (not normally distributed). The Shapiro–Wilk test was used to test the normality of the data. Categorical variables are reported as numbers and percentages. Univariate and multivariate Cox proportional hazard models were used to identify predictors of 90-day mortality, based on clinical risk factors. All predictors which were statistically significant (*p* < 0.05) were used to adjust the outcome analysis. The Cox-adjusted survival curve was used to compare survival between vasoplegic and non-vasoplegic patients. Univariate and multivariate logistic regression analyses were performed to identify independent predictors of vasoplegia.

## 3. Results

### 3.1. Demographics and Preoperative Clinical Status

Between August 2010 and December 2023, 307 patients underwent cfLVAD implantation. Ninety-day follow-up was complete in 99% of the patients; two patients were lost to follow-up. The mean age was 58 years, and 82% of the patients were male. Twenty-three percent of the patients had undergone previous cardiac surgery, primarily coronary artery bypass grafting. Ischemic cardiomyopathy was the cause of HF in half of the patients. The most non-ischemic cause of HF was idiopathic dilated cardiomyopathy (Table 1).

Almost half of the patients were in the Interagency Registry for Mechanically Assisted Circulatory Support (INTERMACS) 4 profile, one-third of the patients were classified as INTERMACS 1 status, and almost all of the patients in the INTERMACS 1 profile were on mechanical circulatory support prior to surgery. Preoperatively, invasive ventilation was required in 17% and vasopressor support in 19% of all patients. Inotropic support with dobutamine, epinephrine, or milrinone (at any dose) was required in 41% of patients (Table 2).

### 3.2. Operative Characteristics

Operative parameters of the cohort are shown in Table 3. Isolated LVAD implantation was performed in 83% of patients. The HeartWare Ventricular Assist Device^TM^ (Medtronic, Minneapolis, MN, USA) was the most commonly implanted device, whereas more than one-third of the patients received a HeartMate 3™ (Abbott, Abbott Park, IL, USA) cfLVAD. Ninety-four percent of patients were operated on with the use of CPB with a mean CBP time of 80 min. Intraoperative hemoadsorption was applied in 71% of the cases; in other cases, the hemoadsorption device was not available or the operation was performed without CPB (18 patients). Among operations without CPB, eleven patients were on veno-arterial extracorporeal membrane oxygenation, one patient was treated with the percutaneous microaxial assist device Impella^®^ (Abiomed Inc., Danvers, MA, USA), and one with the Levitronix™ CentriMag (Abbott, Chicago, IL, USA) biventricular assist device. Five patients underwent surgery without CPB or mechanical circulatory support.

### 3.3. Predictors of 90-Day Mortality

Cox proportional hazard model identified dialysis-dependency, previous cardiac surgery, INTERMACS 1 profile, preoperative inotropic, vasopressor, invasive ventilation and mechanical circulatory support, concomitant right ventricular assist device (RVAD), CPB > 120 min, and vasoplegia as predictors of 90-day mortality. After multivariate analysis, dialysis-dependency, preoperative inotropic support, concomitant RVAD and vasoplegia remained statistically significant (Table 4). These variables were used to adjust for the association between vasoplegia and short-term mortality.

### 3.4. Vasoplegia

**Figure 1:**
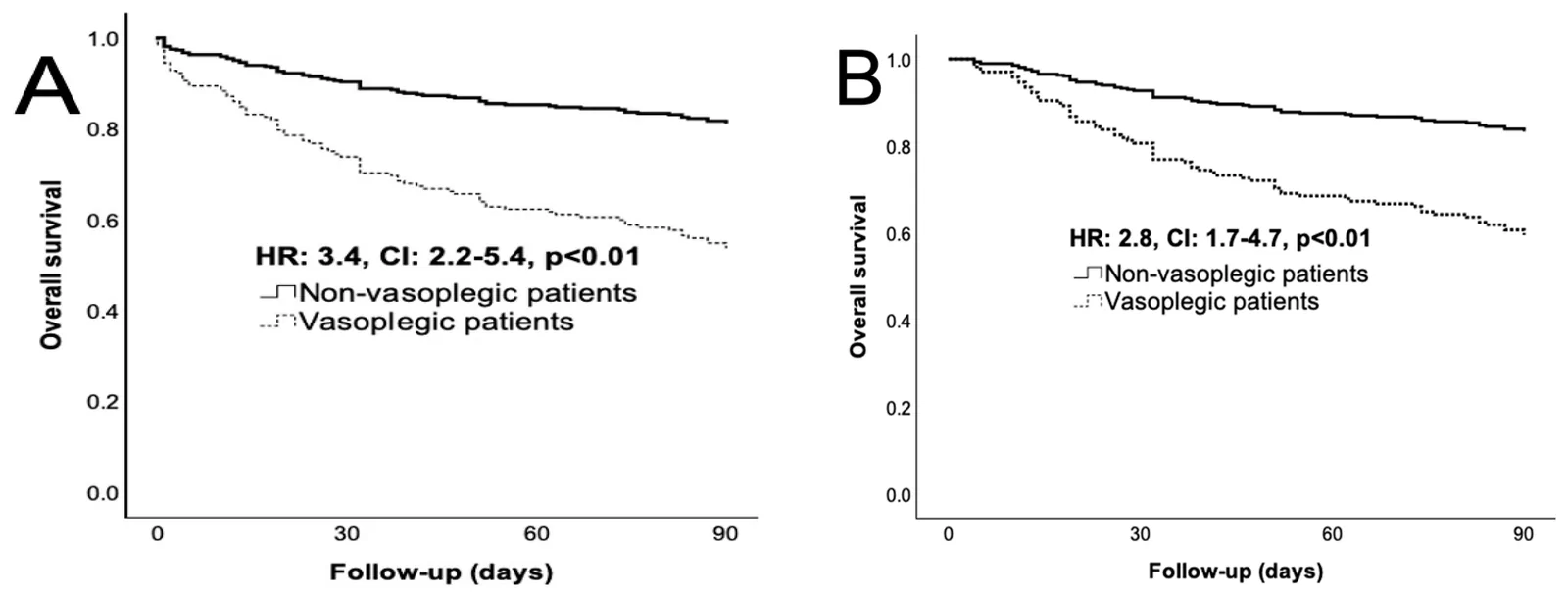
Cox-adjusted survival curves of postoperative vasoplegic (dotted line) and non-vasoplegic (straight line) cfLVAD patients: all patients (**A**); from 72 h landmark (**B**).

Postoperative vasoplegia occurred in 70 patients, resulting in an incidence of 23%. The 30-day and 90-day all-cause mortality was 16% and 26%, respectively (Table 5). The 30-day mortality was 40% in vasoplegic patients versus 8% in non-vasoplegic patients. The 90-day mortality was 50% in vasoplegic patients versus 19% in non-vasoplegic patients. After adjustment for dialysis-dependency, preoperative inotropic support and concomitant RVAD implantation, vasoplegic patients had significantly higher adjusted 30-day (HR: 4.9, CI: 2.7–8.9, *p* < 0.01) and 90-day (HR: 3.4, CI: 2.2–5.4, *p* < 0.01) mortality compared to non-vasoplegic patients (Figure 1A). To adjust for immortal time bias, the analysis was repeated from the 72 h landmark, excluding 15 patients. The result showed slightly lower association between vasoplegia and 90-day mortality (HR: 2.8, CI: 1.7–4.7, *p* < 0.01) (Figure 1B).

Postoperative vasoplegic patients were significantly more likely to require mechanical RV support, RRT and extended antibiotic therapy due to infection. RV failure and infection are possible confounders of the current vasoplegia definition. Therefore, we performed a sensitivity analysis excluding all patients requiring any RVAD or with infection. The association between vasoplegia and 90-day mortality remained strong and significant after excluding RVAD patients (HR: 2.9, CI: 1.7–5.0, *p* < 0.01) and after excluding infection patients (HR: 4.1, CI: 2.2–7.9, *p* < 0.01). ICU stay was longer in vasoplegic patients than in non-vasoplegic patients but did not reach statistical significance. Hospital stay was non-significantly shorter in vasoplegic patients, explained by death as competing risk.

Univariate logistic regression analysis (Table 6) showed preoperative dialysis-dependency, INTERMACS 1 profile, preoperative vasopressor support, preoperative invasive mechanical ventilation and preoperative mechanical circulatory support as significant risk factors for the occurrence of postoperative vasoplegia. After multivariate analysis, preoperative vasopressor support remained the only independent clinical predictor for the occurrence of vasoplegia in the postoperative course (OR: 2.0, CI: 1.0–4.0, *p* = 0.04). Preoperative invasive ventilation remained a clinically significant predictor of vasoplegia (OR: 2.2, CI: 1.0–5.0, *p* = 0.05) but did not reach statistical significance. Intraoperative hemoadsorption reduced the risk of postoperative vasoplegia and remained statistically significant in the multivariate regression analysis (OR: 0.5, CI: 0.3–0.5, *p* = 0.03).

## 4. Discussion

Postoperative vasoplegia after cardiac surgery remains a serious complication, especially in high-risk patients such as HF patients. In the present analysis, we found that almost a quarter of patients who underwent cfLVAD implantation developed relevant postoperative vasoplegia. Furthermore, vasoplegia was significantly associated with increased short-term mortality. Preoperative vasopressor support or preoperative invasive mechanical ventilation were significant independent predictors for the development of postoperative vasoplegia, whereas the use of intraoperative hemoadsorption was significantly associated with the prevention of postoperative vasoplegia in patients undergoing cfLVAD implantation.

The incidence of postoperative vasoplegia after cfLVAD implantation has been studied in small cohorts. Van Vessem et al. evaluated the incidence and outcome of vasoplegia after HF surgery [12]. The incidence of vasoplegia was 60% among patients undergoing LVAD implantation. However, the LVAD population consisted of only 33 patients (15% of the study population). Tecson et al. reported a slightly lower (49%) incidence of postoperative vasoplegia after cfLVAD implantation [13]. It should be noted that the definition used in their analysis was according to the number of vasopressors required and did not specify the dosage. In another retrospective study, de Waal et al. reported that 33% of the cfLVAD population developed postoperative vasoplegia [14]. Although their definition of vasoplegia in this study was comparable, the overall population consisted of a combination of short- and long-term cfLVAD.

The reported incidence of vasoplegia after cfLVAD implantation varies substantially due to differences in definition, patient characteristics and perioperative management. The current literature lacks a standardized definition for postoperative vasoplegia, often relying on complex criteria. Most studies incorporate vasopressor dosage and the number of agents required to maintain a target mean arterial pressure (typically 50–65 mmHg) during the early postoperative phase (24–72 h) over a specified duration (3–12 h). Hemodynamic variables, such as cardiac index and systemic vascular resistance index, are frequently included, with norepinephrine or its equivalent utilized as the primary vasopressor. While a threshold dose of 0.2 µg/kg/min is widely accepted to define vasoplegia, the required duration of this dosage varies substantially across studies. We propose that a minimum 12 h threshold is necessary to account for early transient complications, such as metabolic and coagulation disorders, thereby excluding other transient causes of high vasopressor requirements. Notably, specific exclusion of RV failure is not included in most definitions of vasoplegia. However, fluid resuscitation in vasoplegic patients relies on different hemodynamic parameters, including RV function. Although hemodynamic parameters like cardiac and systemic vascular resistance indices enhance specificity, they exhibit high temporal variability and depend on the presence of a pulmonary artery catheter, which is not universally utilized. Furthermore, differentiating early vasoplegia from postoperative sepsis remains challenging; inflammatory markers routinely elevate following cardiac surgery, and blood culture results are inherently delayed. Consequently, antimicrobial escalation at our institution is guided by the overall clinical trajectory rather than isolated laboratory or culture data. Therefore, our simplified definition—which couples vasopressor requirements with objective clinical endpoints, such as severe bleeding or tamponade requiring surgical revision—provides a pragmatic, highly relevant approach for clinical practice.

The association between vasoplegia and adverse outcomes, including increased mortality, is well-established across the literature. Consequently, identifying reliable predictors of postoperative vasoplegia is critical for developing effective preventive strategies. Previous studies have identified several risk factors, ranging from medical history and laboratory biomarkers to preoperative clinical status. Notably, a preoperative requirement for vasopressor support and/or mechanical ventilation serves as a strong clinical predictor of vasoplegia. These findings raise the clinical question of whether early surgical intervention or optimized preoperative stabilization—utilizing mechanical circulatory support and targeted pulmonary therapy—could mitigate this risk and improve patient outcomes.

Postoperative vasoplegia after LVAD implantation is a result of an inflammatory response which is regulated by circulating cytokines [15]. Among the studied cytokines, tumor necrosis factor alpha (TNF-α), interleukin 6 (IL-6) and IL-8 are of particular interest in HF and LVAD patients [16]. Preoperatively, proinflammatory cytokines such as TNF-α and IL-6 are elevated in HF patients and higher levels of these cytokines are associated with increased mortality [17]. A postoperative increase in these cytokines is associated with poor outcomes [18,19]. Therefore, it is plausible that removal of cytokines by intraoperative hemoadsorption is a possible preventive measure against postoperative vasoplegia in patients undergoing LVAD implantation. Furthermore, laboratory abnormalities such as anemia and hyperthyroidism should be corrected [11]. Although difficult to achieve in heart failure patients, recovery from renal failure should be aimed for, as reduced creatinine clearance or dialysis-dependency are possible risk factors.

## 5. Limitations

Although this is a single-center retrospective cohort study spanning 13 years, with major device and medical management changes over time, it remains a large cohort reporting on the incidence and outcomes of postoperative vasoplegia in patients undergoing cfLVAD implantation. Furthermore, due to the structured and almost complete follow-up, the results are plausible and could be translated into clinical practice. The selected characteristics for regression analysis were chosen for their clinical relevance. However, the statistical results still depend on the chosen criteria and the characteristics of the studied population. Therefore, this remains a potential limitation of this study. Furthermore, due to incomplete hemodynamic and cytokine data, the role of RV failure and hemoadsorption on the current vasoplegia definition remain uncertain. Future studies are needed to identify predictors and potential preventive measures of vasoplegia in patients undergoing cfLVAD implantation to improve clinical outcomes in this population.

## 6. Conclusions

Postoperative vasoplegia remains a common and clinically relevant complication following cfLVAD implantation. Preoperative invasive ventilation and vasopressor support are significant predictors of postoperative vasoplegia. Intraoperative hemoadsorption may reduce the risk of postoperative vasoplegia. Future studies are needed to evaluate the role of intraoperative hemoadsorption in patients undergoing cfLVAD implantation.

## Figures and Tables

**Table 1:** Baseline demographics.

Variables	N = 307
Age, *years ± SD*	58 ± 11
Male gender, *n (%)*	252 (82)
LVEF, *% ± SD*	16 ± 7
Atrial fibrillation, *n (%)*	113 (37)
COPD, *n (%)*	55 (18)
Dialysis dependence, *n (%)*	41 (13)
Previous cardiac surgery, *n (%)*	69 (23)
Previous CABG, *n (%)*	56 (18)
Ischemic cardiomyopathy, *n (%)*	152 (50)
Dilated cardiomyopathy (idiopathic), *n (%)*	123 (40)
Other cardiomyopathies, *n (%)*	32 (10)

Data are presented as mean ± SD or number (percentage). LVEF, left ventricular ejection fraction; COPD, chronic obstructive pulmonary disease; CABG, coronary artery bypass grafting; SD, standard deviation.

**Table 2:** Preoperative clinical status.

Variables	N = 307
INTERMACS 1, *n (%)*	99 (32)
INTERMACS 2, *n (%)*	23 (7)
INTERMACS 3, *n (%)*	27 (9)
INTERMACS 4, *n (%)*	146 (48)
Preoperative inotropic support, *n (%)*	127 (41)
Preoperative vasopressor support, *n (%)*	57 (19)
Preoperative invasive ventilation, *n (%)*	51 (17)
Preoperative MCS, *n (%)*	97 (32)
VA-ECMO	56/97
IABP	12/97
Percutaneous microaxial assist device	14/97
VA-ECMO and percutaneous microaxial assist device	6/97
Biventricular assist device	9/97

Data are presented as median (interquartile range) or number (percentage). INTERMACS, Interagency Registry for Mechanically Assisted Circulatory Support; MCS, mechanical circulatory support; VA-ECMO, veno-arterial extracorporeal membrane oxygenation; IABP, intra-aortic balloon pump.

**Table 3:** Operative characteristics.

Variables	N = 307
Isolated LVAD implantation, *n (%)*	255 (83)
HVAD, *n (%)*	193 (63)
HM3, *n (%)*	114 (37)
Concomitant procedure, *n (%)*	52 (17)
RVAD, *n (%)*	19 (6)
AV replacement, *n (%)*	15 (5)
TV repair, *n (%)*	12 (4)
Aortic repair, *n (%)*	3 (1)
Other, *n (%)*	3 (1)
CPB time, *minutes (IQR)*	80 (65–105)

Data are presented as numbers (percentages) or median (IQR). LVAD, left ventricular assist device; HVAD, HeartWare Ventricular Assist Device; HM3, Heart Mate 3; RVAD, right ventricular assist device; AV, aortic valve; TV, tricuspid valve; CPB, cardiopulmonary bypass; IQR, interquartile range.

**Table 4:** Predictors of 90-day mortality.

Variables	Univariate Analysis		Multivariate Analysis	
	HR (95% CI)	*p*	HR (95% CI)	*p*
Dialysis-dependency	2.8 (1.7–4.5)	<0.01	1.9 (1.0–3.3)	0.04
Previous cardiac surgery	1.6 (1.0–2.6)	0.05	1.4 (0.8–2.3)	0.24
INTERMACS 1	2.0 (1.3–3.1)	0.02	3.3 (0.4–28.7)	0.28
Preoperative poor RV function	1.5 (0.8–2.8)	0.17		
Preoperative inotropic support	2.4 (1.5–3.7)	<0.01	1.9 (1.1–3.2)	0.02
Preoperative vasopressor support	2.0 (1.3–3.3)	<0.01	1.1 (0.6–1.9)	0.78
Preoperative invasive ventilation	2.0 (1.2–3.3)	<0.01	0.9 (0.5–1.7)	0.72
Preoperative MCS	1.9 (1.2–3.0)	<0.01	0.3 (0.0–2.7)	0.28
Concomitant procedure	2.2 (1.3–3.5)	<0.01	1.2 (0.6–2.6)	0.60
Concomitant RVAD implantation	4.1 (2.2–7.5)	<0.01	2.5 (1.1–5.8)	0.03
Intraoperative hemoadsorption	0.7 (0.5–1.1)	0.13		
CPB > 120 min	1.9 (1.1–3.1)	0.01	1.5 (0.8–2.8)	0.18
Vasoplegia	3.4 (2.2–5.4)	<0.01	2.9 (0.8–2.8)	<0.01

Data are presented as Hazard Ratio (95% Confidence Interval). INTERMACS, Interagency Registry for Mechanically Assisted Circulatory Support; RV, right ventricle; MCS, mechanical circulatory support; RVAD, right ventricular assist device; CPB, cardiopulmonary bypass.

**Table 5:** Outcomes.

Variables	All Patients N = 307	Vasoplegic N = 70	Non-Vasoplegic N = 237	HR (95% CI)	*p*
**Primary endpoints**					
Vasoplegia, *n (%)*	70 (23)				
30-day mortality, *n (%)*	48 (16)	28 (40)	20 (8)	4.9 (2.7–8.9)	<0.01
90-day mortality, *n (%)*	81 (26)	35 (50)	46 (19)	3.0 (1.9–4.7)	<0.01
**Secondary endpoints**					
Mechanical RV support, *n (%)*	24 (8)	12 (17)	12 (5)	4.3 (1.5–12.6)	<0.01
Stroke, *n (%)*	27 (9)	7 (10)	20 (8)	1.2 (0.5–3.1)	0.65
Dialysis, *n (%)*	83 (27)	26 (37)	57 (24)	2.5 (1.4–4.7)	<0.01
Prolonged ventilation, *n (%)*	81 (26)	23 (33)	58 (24)	1.4 (0.8–2.5)	0.30
Infection, *n (%)*	123 (40)	39 (56)	84 (35)	2.1 (1.2–3.6)	0.01
ICU stay, *days (IQR)*	15 (7–33)	19 (9–41)	14 (7–31)	NA	0.25
Hospital stay, *days (IQR)*	25 (14–41)	23 (11–41)	27 (14–42)	NA	0.52

Data are presented as number (percentage) or median (interquartile range). RV, right ventricle; ICU, intensive care unit; IQR, interquartile range.

**Table 6:** Predictors of vasoplegia.

Variables	Univariate Analysis		Multivariate Analysis	
	OR (95% CI)	*p*	OR (95% CI)	*p*
Dialysis dependence	2.9 (1.4–5.7)	0.003	1.8 (0.9–3.9)	0.11
Previous cardiac surgery	1.7 (0.9–3.1)	0.088		
INTERMACS 1	2.1 (1.2–3.7)	0.007	3.2 (0.2–64.6)	0.45
Preoperative poor RV function	1.4 (0.6–3.1)	0.409		
Preoperative inotropic support	1.5 (0.9–2.5)	0.165		
Preoperative vasopressor support	2.9 (1.6–5.4)	<0.001	2.0 (1.0–4.0)	0.04
Preoperative invasive ventilation	3.3 (1.7–6.2)	<0.001	2.3 (1.0–5.1)	0.05
Preoperative MCS	2.1 (1.2–3.6)	0.009	0.3 (0.0–6.3)	0.45
Concomitant procedure	1.0 (0.5–2.1)	0.959		
Concomitant RVAD implantation	2.1 (0.8–5.5)	0.139		
Intraoperative hemoadsorption	0.4 (0.3–0.7)	0.003	0.5 (0.3–0.9)	0.03
CPB > 120 min	1.6 (0.8–3.1)	0.147		

Data are presented as Odds Ratio (95% Confidence Interval). INTERMACS, Interagency Registry for Mechanically Assisted Circulatory Support; RV, right ventricle; MCS, mechanical circulatory support; RVAD, right ventricular assist device; CPB, cardiopulmonary bypass.

## Data Availability

Data underlying this article will be shared on reasonable request.
